# Gender Differences in Neurodegeneration, Neuroinflammation and Na^+^-Ca^2+^ Exchangers in the Female A53T Transgenic Mouse Model of Parkinson’s Disease

**DOI:** 10.3389/fnagi.2020.00118

**Published:** 2020-05-07

**Authors:** Giulia Costa, Maria Jose Sisalli, Nicola Simola, Salvatore Della Notte, Maria Antonietta Casu, Marcello Serra, Annalisa Pinna, Antonio Feliciello, Lucio Annunziato, Antonella Scorziello, Micaela Morelli

**Affiliations:** ^1^Department of Biomedical Sciences, Section of Neuroscience, University of Cagliari, Cagliari, Italy; ^2^Department of Neuroscience, Reproductive and Odontostomatological Sciences, University of Naples Federico II, Naples, Italy; ^3^National Institute of Neuroscience (INN), University of Cagliari, Cagliari, Italy; ^4^National Research Council of Italy, Institute of Translational Pharmacology, UOS of Cagliari, Scientific and Technological Park of Sardinia POLARIS, Pula, Italy; ^5^National Research Council of Italy, Neuroscience Institute, Cagliari, Italy; ^6^Department of Molecular Medicine and Medical Biotechnologies, University of Naples Federico II, Naples, Italy; ^7^SDN Research Institute Diagnostics and Nuclear (IRCCS SDN), Naples, Italy

**Keywords:** constipation, dopamine, GFAP, IBA-1, memory, midbrain, NCXs, striatum

## Abstract

Twelve-month-old male mice expressing the human A53T variant of α-synuclein (A53T) develop dopamine neuron degeneration, neuroinflammation, and motor deficits, along with dysfunctions of the mitochondrial Na^+^-Ca^2+^ exchanger (NCX) isoforms 1 (NCX1) and 3 (NCX3) in the nigrostriatal system. Since gender is thought to play a role in the etiology of Parkinson’s disease (PD), we characterized neurochemical and behavioral alterations in 12-month-old female A53T transgenic mice. We investigated the presence of dopaminergic degeneration, astrogliosis and microgliosis using immunohistochemistry for tyrosine hydroxylase (TH), glial fibrillary acidic protein (GFAP) and ionized calcium-binding adaptor molecule-1 (IBA-1) in both the substantia nigra pars compacta (SNc) and striatum. In the same regions, we also evaluated the co-localization of NCX1 in cells positive for IBA-1 and the co-localization of NCX3 in TH-positive neurons and fibers. Furthermore, in both male and female mice, we performed motor (beam walking and pole tests) and memory [novel object recognition (NOR) and spontaneous alternation] tasks, together with tests to evaluate peripheral deficits (olfactory and stool collection tests). Female A53T transgenic mice displayed degeneration of nigral dopaminergic neurons, but neither microgliosis nor astrogliosis in the SNc and striatum. Moreover, female A53T transgenic mice displayed co-localization between NCX1 and IBA-1 positive cells in the striatum but not SNc, whereas NCX3 did not co-localize with either TH-positive terminals or neuronal bodies in the nigrostriatal system. Furthermore, female A53T transgenic mice showed increased crossing time in the beam walking test, but no impairments in the pole or memory tests, and in tests that evaluated peripheral deficits, whereas male A53T transgenic mice displayed motor, memory and peripheral deficits. Immunohistochemical and behavioral results obtained here in the female mice differ from those previously observed in males, and suggest a dissimilar influence of NCX1 and NCX3 on dopaminergic function in female and male A53T transgenic mice, strengthening the validity of these mice as a model for studying the etiological factors of PD.

## Introduction

Parkinson’s disease (PD) is a complex neurodegenerative disorder characterized by the demise of dopaminergic neurons in the substantia nigra pars compacta (SNc) and by the reduction of dopaminergic tone at the level of the striatum (Obeso et al., 2010, 2017; Halliday et al., 2011; Costa and Morelli, 2015). Although most patients develop idiopathic PD (Mayeux et al., 1995), several factors, such as gender, mutations in specific genes, neuroinflammation, mitochondrial dysfunctions, oxidative stress, excitotoxicity and dysfunction of the protein degradation system have been shown to increase the likelihood of developing PD (Schapira and Jenner, 2011; Pang et al., 2019).

In particular, gender seems to be a key factor in PD, since several studies have demonstrated that men have a two-fold (or higher) increase in the relative risk of developing the disease compared to women, irrespective of age (Baldereschi et al., 2000; Gillies et al., 2014; Labandeira-Garcia et al., 2016). Notably, differences in sex hormones, particularly estrogens, do not adequately account for the influence of gender on the manifestation of PD, as shown by the contrasting results obtained in studies that evaluated the relationship between the use of postmenopausal hormone therapy and variations in the risk of developing PD in women (Simon et al., 2009). Thus, a possible explanation for such a gender effect may be provided by the evidence that intrinsic differences exist between the brains of men and women in structures that are affected by PD. For example, it has been shown that men express higher numbers of neuronal cells and regulatory networks in the nigrostriatal dopaminergic system, compared to women (Gillies and McArthur, 2010; Villa et al., 2016). Another study found a dissimilar upregulation in the expression of genes related to familial forms of PD in parkinsonian men and women (Cantuti-Castelvetri et al., 2007). More specifically, an upregulation of genes involved in signal transduction and neuronal maturation was observed in parkinsonian women, while an upregulation of genes implicated in the pathogenesis of PD (α-synuclein and PINK1) was found in parkinsonian men (Cantuti-Castelvetri et al., 2007). Another possible explanation for the gender effect observed in PD may involve alterations in mitochondrial function (Briston and Hicks, 2018; Reeve et al., 2018; McAvoy and Kawamata, 2019), and in particular an impairment in complex I of the electron transport system. In this regard, one study that genotyped the polypeptides encoded by the mitochondrial genome in both PD patients and general population found that the single-nucleotide polymorphism 10398G seemed to be associated with a decreased risk of PD and that this association appeared to be stronger in women than in men (van der Walt et al., 2003). Moreover, the same study found that single-nucleotide polymorphism 9055A was associated with a reduced risk of PD in women, but not in men (van der Walt et al., 2003).

In the context of preclinical studies on familial forms of PD, transgenic mice expressing the human A53T variant of α-synuclein have emerged as a valuable experimental model. From 8 months of age, A53T transgenic mice display inclusions of α-synuclein, a pathological hallmark of PD, with dense accumulation in the spinal cord, brainstem, cerebellum, and thalamus, whose manifestation parallels the onset of motor impairment (Giasson et al., 2002). Moreover, A53T transgenic mice display mitochondria with abnormal morphology, along with deficiency of mitochondrial complex IV in spinal cord homogenates and damage of mitochondrial DNA in the brainstem, neocortex, and spinal cord ventral horn (Giasson et al., 2002; Martin et al., 2006), thus supporting previous evidence that has implicated mitochondrial dysfunctions in the etiology of PD (Briston and Hicks, 2018; Reeve et al., 2018; McAvoy and Kawamata, 2019). Na^+^-Ca^2+^ exchangers (NCXs) regulate Na^+^ and Ca^2+^ homeostasis and exist in two isoforms, 1 and 3 (Canitano et al., 2003). Both NCX1 and NCX3 are plasma membrane ionic exchangers that can modulate the synthesis and release of neurotransmitters in the central and peripheral nervous systems, as well as the release of anterior pituitary hormones (Annunziato et al., 2004). The NCX1 isoform is expressed in several peripheral organs and the brain (Annunziato et al., 2004); although the exact physiological roles of NCX1 in the brain have not yet been defined, *in vitro* studies have provided evidence that NCX1 is the most highly expressed isoform in microglia (Quednau et al., 1997; Newell et al., 2007; Boscia et al., 2009). The NCX3 isoform is selectively expressed in the brain and skeletal muscle (Papa et al., 2003), where it plays a fundamental role in buffering the intracellular Ca^2+^ and Na^+^ overload that occurs under physiological and pathological conditions (Condrescu et al., 1995; Linck et al., 1998; Secondo et al., 2007). Moreover, NCX3 is also localized on the outer mitochondrial membrane where it conributes to the regulation of mitochondrial Ca^2+^ homeostasis (Scorziello et al., 2013).

In a recent study, we have demonstrated that 12-month-old male A53T transgenic mice display several abnormalities reminiscent of human PD such as the following: (a) decreased immunoreactivity for tyrosine hydroxylase (TH) in the SNc and striatum; (b) increased levels of the neuroinflammatory markers ionized calcium-binding adaptor molecule 1 (IBA-1), in the striatum, and glial fibrillary acidic protein (GFAP), in the SNc and striatum; (c) motor deficits. Moreover, our previous study found that NCX1 was co-expressed in IBA-1-positive microglial cells in the striatum and that NCX3 was co-expressed in TH-positive neurons in the SNc (Sirabella et al., 2018). Taken together, these findings would suggest that mitochondrial dysfunctions dependent on NCXs may be associated with dopamine neuron degeneration and gliosis, both of which may contribute to the PD-like phenotype displayed by male A53T transgenic mice.

The present study was performed to evaluate the gender differences in neurodegeneration, neuroinflammation and NCXs in the nigrostriatal system and glial cells of 12-month-old female A53T transgenic mice, to gain insight into the influence that gender may play in the manifestation of PD-like alterations in this strain of mice. Specifically, we evaluated in both the SNc and striatum the presence of dopaminergic degeneration, astrogliosis and microgliosis using immunoreactivity for TH, GFAP, and IBA-1, respectively. Besides, we evaluated the co-localization of NCX1 in IBA-1-positive cells and of NCX3 in TH-positive fibers and neurons. Finally, we characterized whether male and female A53T transgenic mice displayed a dissimilar performance in a battery of behavioral tasks that included the beam walking and pole tests, used to assess motor performance and motor coordination; the novel object recognition (NOR) and the spontaneous alternation behavior in a Y-maze (SAB) tests, used to evaluate the non-spatial and spatial component of short-term memory; the olfactory and one-hour stool collection tests, used to evaluate the presence of peripheral deficits related to olfactory or intestinal dysfunctions that may precede the occurrence of motor impairment in PD.

## Materials and Methods

### Animals

Twelve-month-old male and female mice expressing the human A53T α-synuclein mutation under the control of a prion promoter (Pmp-SNCA*A53T; Giasson et al., 2002) were obtained from The Jackson Laboratory. Mice hemizygous for the α-synuclein A53T mutation were bred on a mixed C57Bl/6 × C3H background to produce transgenic and non-transgenic littermates. To identify A53T mice, PCR was performed according to the protocol provided by The Jackson Laboratory. All mice were housed in groups of 1–5, in temperature and humidity-controlled rooms under a 12-h light/dark cycle and fed an *ad libitum* diet of standard mouse chow. All experiments were conducted in accordance with the guidelines for animal experimentation of the EU directives (2010/63/EU; L.276; 22/09/2010) and with the guidelines approved by the Ethical Committees of the University of Cagliari and of Federico II University of Naples. Experiments were designed to minimize animal discomfort to the least possible extent and to reduce the number of animals used.

### Immunohistochemistry

#### Tissue Preparation and Staining

Mice were anesthetized and transcardially perfused with paraformaldehyde (4% in 0.1 M phosphate buffer, pH 7.4). For each immunohistochemical evaluation, three sections of 50 μm from the SNc (A: −2.92 mm; −3.28 mm; −3.64 mm from bregma) and striatum (A: 1.10 mm; 0.74 mm; 0.38 mm from bregma), according to the mouse brain atlas of Paxinos and Franklin (2008), were cut coronally on a vibratome. Then, sections were incubated overnight at 4°C with the primary antibody (polyclonal mouse anti-TH, 1:1,000, Sigma–Aldrich, Milan, Italy; monoclonal mouse anti-GFAP, 1:400, Sigma-Aldrich, Milan, Italy; polyclonal goat anti-IBA-1, 1:1,000, Novus Biologicals Europe, Abingdon, UK). Moreover, to investigate NCX1 and NCX3 co-localization in dopaminergic neurons and microglial cells, double immunostaining for IBA-1+NCX1 and TH+NCX3 was performed in the SNc and striatum (polyclonal rabbit anti-NCX1, 1:5,000, Swant, Marly, Switzerland; polyclonal rabbit anti-NCX3, 1:5,000, Swant, Marly, Switzerland). For diaminobenzidine visualization of TH in the SNc, a biotinylated goat anti-mouse immunoglobulin G (IgG; 1:500) was used as a secondary antibody and the avidin-biotin-peroxidase (ABC) complex protocol was followed (Costa et al., 2013). For the visualization of TH, in the striatum, and of GFAP and IBA-1, in the SNc and striatum, the proper fluorescent secondary antibody (AlexaFluor 488-labeled donkey anti-mouse IgG for TH and GFAP; AlexaFluor 594-labeled donkey anti-goat IgG for IBA-1, 1:400) was used (Costa et al., 2018). For the double immunostaining of IBA-1, TH, NCX1, and NCX3, AlexaFluor 594-labeled donkey anti-goat IgG, AlexaFluor 488-labeled donkey anti-mouse IgG, and AlexaFluor 594-labeled donkey anti-rabbit IgG (all 1:400) were used as secondary antibodies. To allow visualization of cell nuclei in the fluorescent staining, sections were finally incubated for 10 min with 4′,6-diamidine-2′-phenylindole dihydrochloride (DAPI, 1:10,000). The sections were mounted on gelatin-coated slides, dehydrated, and mounted on coverslips. Standard control experiments were performed by omission of the primary or secondary antibody and yielded no cellular labeling (data not shown).

#### Stereological Counting of TH-Immunoreactive Neurons in the SNc

Stereological analysis of the total number of TH-positive neurons in the SNc was carried out blind in both hemispheres, using a software (Stereologer) linked to a motorized stage on a light microscope (Pinna et al., 2016; Costa et al., 2019b). The SNc region was outlined at low magnification (2×), and quantification of cells was achieved using automatically randomized sampling and an optical dissector (50 × 50 × 15 μm). Cells were sampled with a 40× objective through a defined depth with a guard zone of 2 μm. Coefficients of error ranged from 0.05 to 0.1 (Costa et al., 2017, 2019a).

#### Analysis of TH Immunoreactivity in the Striatum

Images were digitized (Axio Scope A1, Zeiss, Oberkochen, Germany) in greyscale and captured at 5× magnification. Analysis was performed in a blinded manner in the three sections. The density of immunoreacted fibers was determined quantitatively using the ImageJ software (U.S. National Institutes of Health, Bethesda, MD, USA). The final values are expressed as a percentage of the WT group. No significant differences in the densities of immunoreacted fibers were found among the three sections (data not shown); accordingly, values from different levels were averaged.

#### Analysis of GFAP- and IBA-1-Positive Cells in the SNc and Striatum

In each of the three brain sections the whole SNc and two portions of the striatum (dorsolateral and ventromedial), left and right, were acquired with the same epifluorescence microscope cited above. The sections were captured at 10× magnification for analysis of the SNc, or 20× magnification for analysis of the striatum. The number of cells labeled with the nuclear marker DAPI was counted manually for each level of the SNc and striatum using the ImageJ software. Cells were counted when a cell body with branching processes was observed, as well as when processes were detected that converged onto a central point, likely corresponding to the position of a cell body deeper in the tissue. GFAP-/IBA-1-expressing fibers without a clear indication of associated cell bodies were not counted. To assure that the quantification of the number of GFAP-/IBA-1-positive cells in a single section accurately reflected the total number of GFAP-/IBA-1-positive cells, we analyzed only those cells labeled with the nuclear marker DAPI.

#### Analysis of TH+NCX3 and IBA-1+NCX1 Co-localization in the SNc and Striatum

Each of the three brain sections, (i.e., whole SNc, dorsolateral and ventromedial striatum, left and right) were acquired at high magnification (40×) using an epifluorescence microscope as described above. Quantitative analysis of co-localization of TH with NCX3 and of IBA-1 with NCX1 was conducted using the ImageJ plugin Just Another Co-localization Plugin (JACoP; Bolte and Cordelières, 2006). A correlation of signal intensity was calculated as a Pearson correlation coefficient (Rr). The coefficient Rr is a quantitative measurement that estimates the degree of overlap between the fluorescence signals obtained from two channels (Dunn et al., 2011).

### Behavioral Tests

#### Beam Walking Test

The motor performance and motor coordination of female A53T transgenic and WT mice were evaluated with the beam walking test (Ogawa et al., 1985; Fleming et al., 2004; Hwang et al., 2005; Meredith and Kang, 2006; Quinn et al., 2007). In this test, mice were trained to traverse the length of a Plexiglas beam divided into four sections (25 cm each, 1 m total length) with a decreasing width of 4, 3, 2, or 1 cm; the beam was placed on a table such that it led into the mouse home cage. Mice received 2 days of training before testing. On the first day, mice received two assisted trials, involving the placement of each individual mouse on one extremity of the beam to encourage forward movement along the beam. After two assisted trials, mice were able to traverse the entire length of the beam unassisted. The 2-day training sessions ended when all mice completed five unassisted runs across the entire length of the beam. To render the task more challenging, on the test day a mesh grid (1 cm squares) was placed over the beam surface. Mice were videotaped for a total of five trials. An error was counted when a limb slipped through the grid during a forward movement; therefore, every mouse could make a maximum of four slips per step. By scoring each limb slip individually, the severity of errors could be measured. Time to traverse the beam, number of steps, and error per step scores were calculated across all the five trials and averaged for each group.

#### Pole Test

The pole test was used to evaluate the agility of female A53T transgenic and WT mice. This test has been previously used to assess basal ganglia-related motor impairment in mice, since it involves skilled forelimb grasping and maneuvering which require an intact basal ganglia and activation of the rubrospinal pathway (Ogawa et al., 1985, 1987; Matsuura et al., 1997; Sedelis et al., 2001; Fernagut et al., 2003; Fleming et al., 2004). Mice received 2 days of training before testing, during which they were placed head upward at the top of a vertical rough-surfaced pole (diameter 1 cm; height 55 cm). On the test day, the time spent by each mouse to reach the floor was recorded during three trials and the average score was expressed in seconds.

#### Novel Object Recognition (NOR) Task

NOR experiments were performed in male and female A53T and WT mice using a Plexiglas cage (length 25.5 cm, width 19 cm, height 14 cm) with a sawdust-covered floor. Objects to be discriminated were made of plastic and differed in shape and color. Moreover, objects had no genuine significance and had not been previously associated with either rewarding or aversive stimuli. The experimental procedure consisted of three phases: habituation to the test cage for 5 min (S0), acquisition (S1), and testing (S2). Acquisition was performed the day after S0, by placing each mouse in the test cage together with two identical copies of an object (familiar objects). Mice were left to freely explore the objects for 3 min. The testing phase took place 60 min after S1. Mice were exposed to one copy of the objects already presented in S1, plus another object that they had never experienced before (novel object). Object exploration was defined as the mouse sniffing, gnawing, or touching the objects with its nose, whereas sitting on and/or circling the objects were not considered exploratory behaviors. To avoid any olfactory cues, objects were thoroughly cleaned after each session. Moreover, the combination of objects (familiar vs. novel) and their respective locations in the cage (right vs. left) were counterbalanced to prevent biased preferences for specific objects and/or locations. Mice performance was videotaped, and the following parameters were evaluated: (a) total amount of time spent by each mouse exploring the objects during S1 and S2; and (b) percentage of time spent exploring the novel object over the total amount of time spent exploring both objects (novel and familiar) during S2 (Simola et al., 2008; Costa et al., 2014).

#### Spontaneous Alternation Behavior in a Y-Maze (SAB)

Experiments were performed in male and female A53T and WT mice using an apparatus made of black PVC that had three equal arms (length 40 cm, width 11 cm, height 20.5 cm). The arms converged onto a central triangular area, resembling the shape of a Y, and the floor of the maze was covered with sawdust. To avoid any olfactory cues, the maze was thoroughly cleaned, and the sawdust was replaced in between each trial. Mice with no prior experience of the maze were individually placed in the central area and left to explore the whole apparatus freely for a single 8-min trial, during which their performance was videotaped. The percentage of SAB was calculated based on the sequence of arm entries, as reported elsewhere (Costa et al., 2014, 2015).

#### Olfactory Test

Male and female A53T and WT mice were food-deprived for 20 h before testing, which was conducted in a clean plastic cage (length 42 cm, width 24 cm, height 15 cm). Each mouse was individually placed in the center of the cage and had to retrieve an odorous pellet that was buried under the bedding (at a depth of 1 cm). The amount of time required to retrieve the pellet and bite it was measured for each mouse tested (Lehmkuhl et al., 2014).

#### One-Hour Stool Collection Test

Male and female A53T and WT mice were individually placed in a clean cage and monitored throughout the 1-h collection period. Fecal pellets were collected immediately after expulsion and placed in sealed 1.5 ml tubes to avoid evaporation. Tubes were weighed to obtain the wet weight of the stool, which was then dried overnight at 65°C and reweighed to obtain the dry weight. The stool water content was calculated from the difference between the wet and dry stool weights (Li et al., 2006).

### Statistics

Statistical analysis was performed with Statistica for Windows (StatSoft, Tulsa, OK, USA). Differences in the immunoreactivity for TH, GFAP, IBA-1, TH+NCX3 and IBA-1+NCX1, and in the scores obtained in the beam walking and pole tests were analyzed by means of unpaired *t*-test. Differences in the scores obtained in the NOR, SAB, olfactory and stool tests were analyzed by means of two-way (gender × genotype) analysis of variance (ANOVA), followed by Newman–Keuls *post hoc* test. Results were considered significant at *p* < 0.05, and were expressed as mean ± SEM for every analysis performed.

## Results

### Immunohistochemical Modifications in Female 12-Month-Old A53T Transgenic Mice

A previous study by our group (Sirabella et al., 2018) has demonstrated that 12-month-old A53T male mice display a decrease in the immunoreactivity for TH in both the SNc and striatum, an increase in the immunoreactivity for IBA1 in the striatum and for GFAP in the SNc and striatum, along with a co-localization of NCX1 in IBA-1-positive cells in the striatum and of NCX3 in TH-positive neurons in the SNc (Table 1).

**Table 1:** Results obtained in 12-month-old male A53T mice, as compared with WT mice, adapted from Sirabella et al. (2018).

	Effect in SNc	Effect in striatum	
TH immunoreactivity	−	−
IBA-1 immunoreactivity	=	+	
GFAP immunoreactivity	+	+	
TH+NCX3 immunoreactivity	+	=	
IBA-1+NCX1 immunoreactivity	=	+
	Time	Errors	Steps
Beam walking test	+	=	=
	Average time	
	to descend		
Pole test	+		

#### TH Immunoreactivity in the SNc and Striatum

**Figure 1:**
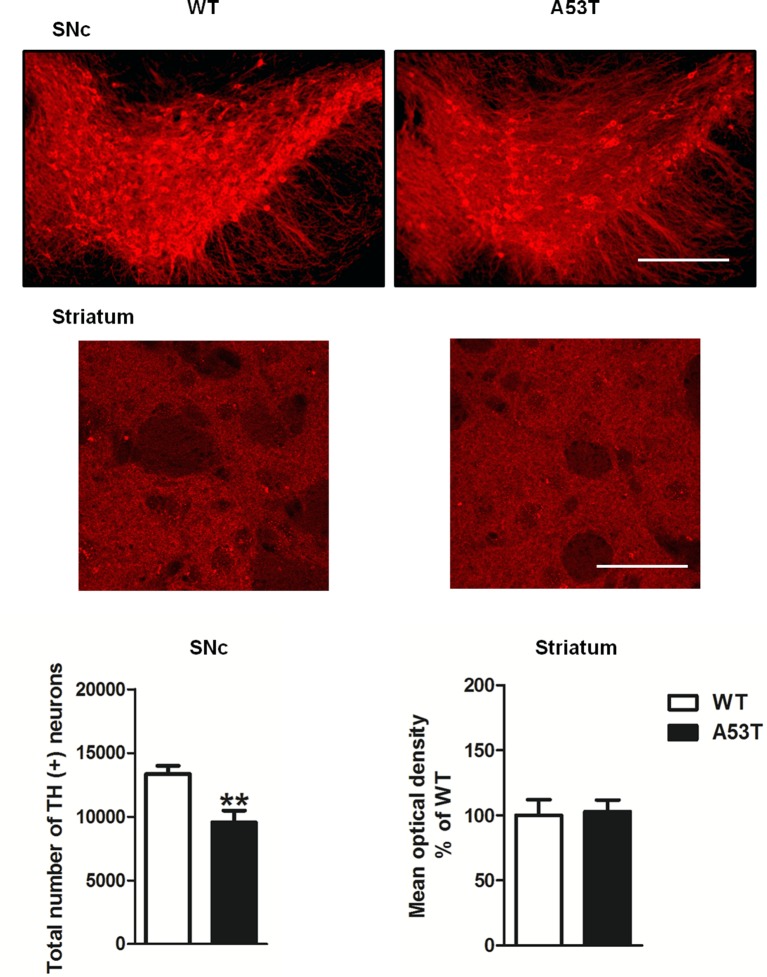
Tyrosine hydroxylase (TH) immunoreactivity in the substantia nigra *pars compacta* (SNc) and striatum of female A53T transgenic and wild type (WT) mice. Representative sections and histograms of the SNc and striatum immunostained for TH of female A53T transgenic and WT mice. The histograms for the SNc show the total number of TH-positive neurons, calculated with stereological analysis, expressed as mean ± SEM. The histograms for the striatum show the density of TH-positive fibers, expressed as mean ± SEM. The number of mice per group is as follows: A53T transgenic mice *n* = 5–17; WT mice *n* = 6–16 for both the SNc and striatum. **p < 0.005 compared with WT mice. Scale bars are 500 μm for the SNc and 50 μm for the striatum.

Female A53T transgenic mice displayed a significant reduction in the total number of TH-positive neurons in the SNc compared with female WT mice (*df* = 9, *t* = 3.469, *p* < 0.01; Figure 1). Conversely, the mean densities of TH-positive fibers in the striatum were comparable between female A53T transgenic mice and female WT mice (*df* = 31, *t* = 0.19, *p* > 0.05; Figure 1).

#### IBA-1 and GFAP Immunoreactivity in the SNc and Striatum

**Figure 2:**
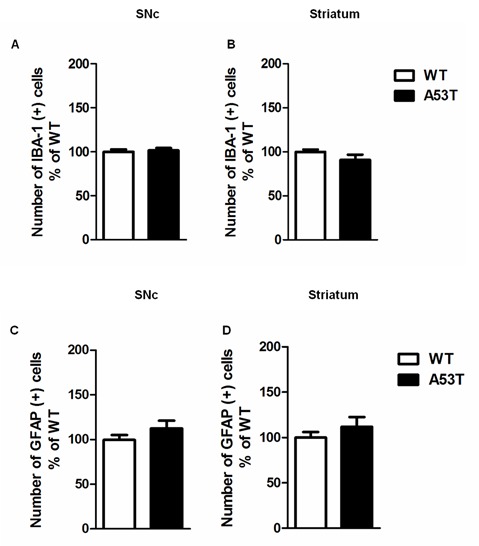
Ionized calcium binding adaptor molecule-1 (IBA-1) and glial fibrillary acidic protein (GFAP) immunoreactivity in the SNc and striatum of female A53T transgenic and WT mice. Representative histograms of the SNc and striatum immunostained for IBA-1 **(A,B)** or GFAP **(C,D)** of female A53T transgenic and WT mice. The histograms show the number of IBA-1- or GFAP-positive cells, expressed as mean ± SEM. The number of mice per group is as follows: A53T transgenic mice *n* = 7; WT mice *n* = 7 for both the SNc and striatum immunostained for IBA-1; A53T transgenic mice *n* = 14; WT mice *n* = 16 for both the SNc and striatum immunostained for GFAP.

Female A53T transgenic mice and female WT mice displayed comparable numbers of cells positive for either IBA-1 or GFAP in the nigrostriatal system. Statistical analysis revealed no significant changes in the immunoreactivity for IBA-1 in the SNc (*df* = 8, *t* = 1.47, *p* > 0.05) and striatum (*df* = 8, *t* = 1.38, *p* > 0.05; Figures 2A,B), as well as no significant changes in the immunoreactivity for GFAP in the SNc (*df* = 28, *t* = 1.32, *p* > 0.05) and striatum (*df* = 28, *t* = 1.02, *p* > 0.05; Figures 2C,D).

#### TH and NCX 3 Co-localization in the SNc and Striatum

**Figure 3:**
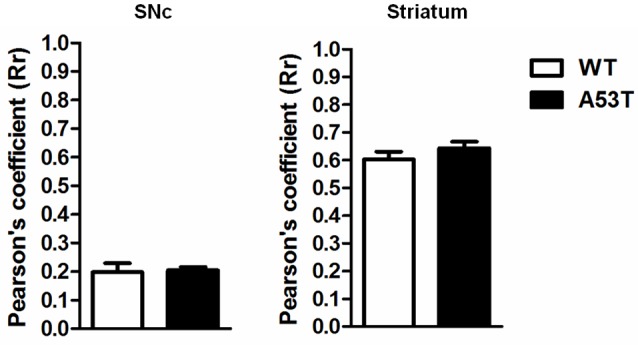
TH+NCX3 co-localization in the SNc and striatum of female A53T transgenic and WT mice. Representative histograms of the SNc and striatum immunostained for TH and Na^+^-Ca^2+^ exchanger 3 (NCX3) of female A53T transgenic and WT mice. The histograms show the values of Pearson’s coefficients, expressed as mean ± SEM. The number of mice per group is: A53T transgenic mice *n* = 7; WT mice *n* = 7 for both the SNc and striatum.

In the SNc and striatum, the co-localization of NCX3 with TH-positive neurons was comparable between female A53T transgenic mice and female WT mice (Figure 3). The average correlation coefficient for the SNc was Rr = 0.133 in female A53T transgenic mice and Rr = 0.119 in female WT mice, and statistical analysis revealed no significant differences between groups (*df* = 8, *t* = 0.19, *p* > 0.05). The average correlation coefficient for the striatum was Rr = 0.644 for female A53T transgenic mice and Rr = 0.604 for female WT mice and statistical analysis revealed no significant differences between groups (*df* = 8, *t* = 1.111, *p* > 0.05).

#### IBA-1 and NCX1 Co-localization in the SNc and Striatum

**Figure 4:**
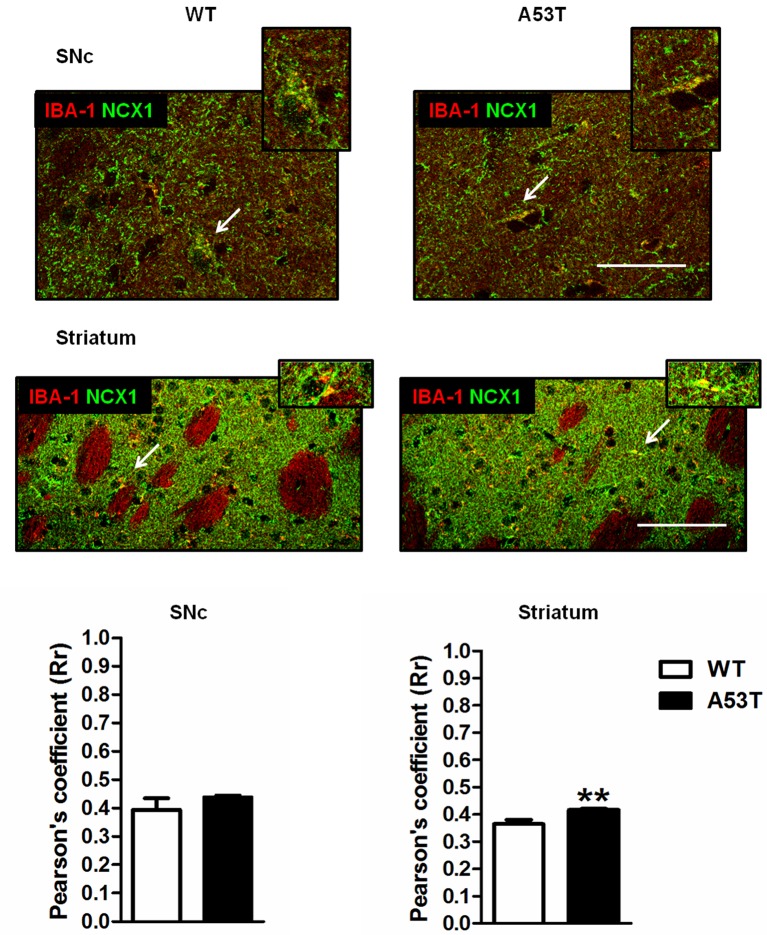
IBA-1 + NCX1 co-localization in the SNc and striatum of female A53T transgenic and WT mice. Representative sections and histograms of the SNc and striatum immunostained for IBA-1 (red) and NCX1 (green) of female A53T transgenic and WT mice. The histograms show the values of Pearson’s coefficients, expressed as mean ± SEM. Arrowheads indicate brain regions where IBA-1 and NCX1 signals co-localized (yellow). The number of mice per group is as follows: A53T transgenic mice *n* = 7; WT mice *n* = 7 for both the SNc and striatum. **p < 0.005 compared with WT mice. The scale bar is 50 μm.

In the SNc, the co-localization of NCX1 with IBA-1-positive cells was comparable between female A53T transgenic mice and female WT mice (Figure 4). The average correlation coefficient for the SNc was Rr = 0.438 for female A53T transgenic mice and Rr = 0.395 for female WT mice; statistical analysis revealed no significant differences between groups (*df* = 8, *t* = 1.07, *p* > 0.05).

Conversely, in the striatum, a more marked co-localization of NCX1 with IBA-1-positive cells was observed in female A53T transgenic mice, compared with female WT mice (Figure 4). The average correlation coefficient for the striatum was Rr = 0.417 for female A53T transgenic mice and Rr = 0.366 for female WT mice; statistical analysis revealed a significant difference between groups (*df* = 8, *t* = 3.494, *p* < 0.01).

### Motor Performance of Female 12-Month-Old A53T Transgenic Mice

#### Beam Walking Test

**Figure 5:**
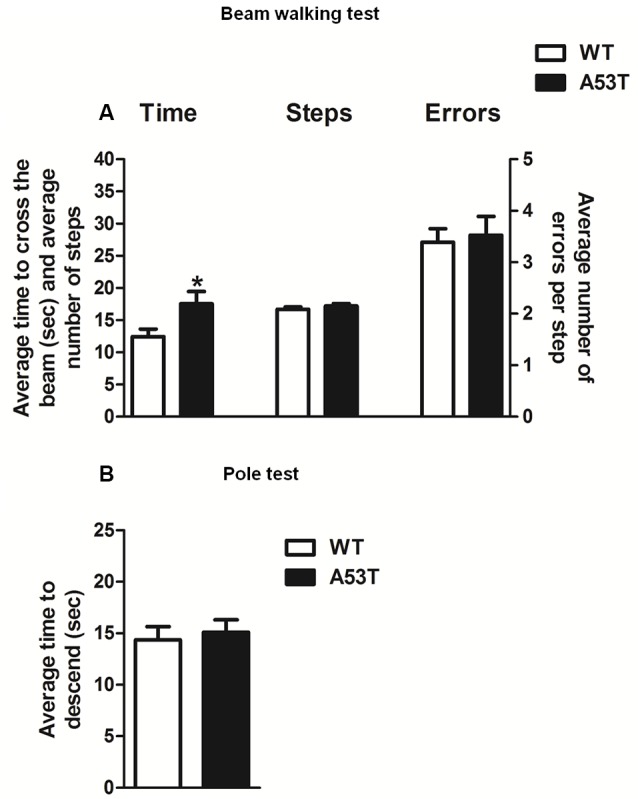
Motor tests in female A53T transgenic and WT mice. Representative histograms for the beam walking test **(A)** and pole test **(B)** evaluated in female A53T transgenic and WT mice. The histogram in **(A)** shows the average time to cross the beam (s), the average number of steps to cross the beam and the average number of errors per step. The histogram in **(B)** shows the average time to descend the pole. Data are expressed as mean ± SEM across five trials in **(A)**, and three trials in **(B)**. The number of mice per group is as follows: A53T transgenic mice *n* = 17; WT mice *n* = 16 for **(A)**, A53T transgenic mice *n* = 10; WT mice *n* = 9 for **(B)**. *p < 0.05 compared with WT mice.

Female A53T transgenic mice required more time to traverse the beam, compared with female WT mice and statistical analysis revealed a significant difference between groups (*df* = 31, *t* = 2.250, *p* < 0.05; Figure 5A). Nevertheless, female A53T transgenic mice and female WT mice performed comparable numbers of steps and errors per step during the test, and statistical analysis revealed no significant differences between groups (number of steps: *df* = 31, *t* = 1.035, *p* > 0.05; errors per steps: *df* = 31, *t* = 0.305, *p* > 0.05; Figure 5A).

#### Pole Test

Female A53T transgenic mice and female WT mice required a comparable amount of time to descend the pole; statistical analysis revealed no significant differences between groups (*df* = 17, *t* = 0.417, *p* > 0.05; Figure 5B).

### Memory and Peripheral Tasks in Male and Female 12-Month-Old A53T Transgenic Mice

Male and female A53T transgenic and WT mice were tested in the NOR and SAB tasks to evaluate memory, as well as in the olfactory and 1-h stool collection tests to evaluate the presence of peripheral deficits. Male, but not female, A53T transgenic mice displayed significant abnormalities in NOR and 1-h stool collection tests.

#### NOR Task

**Figure 6:**
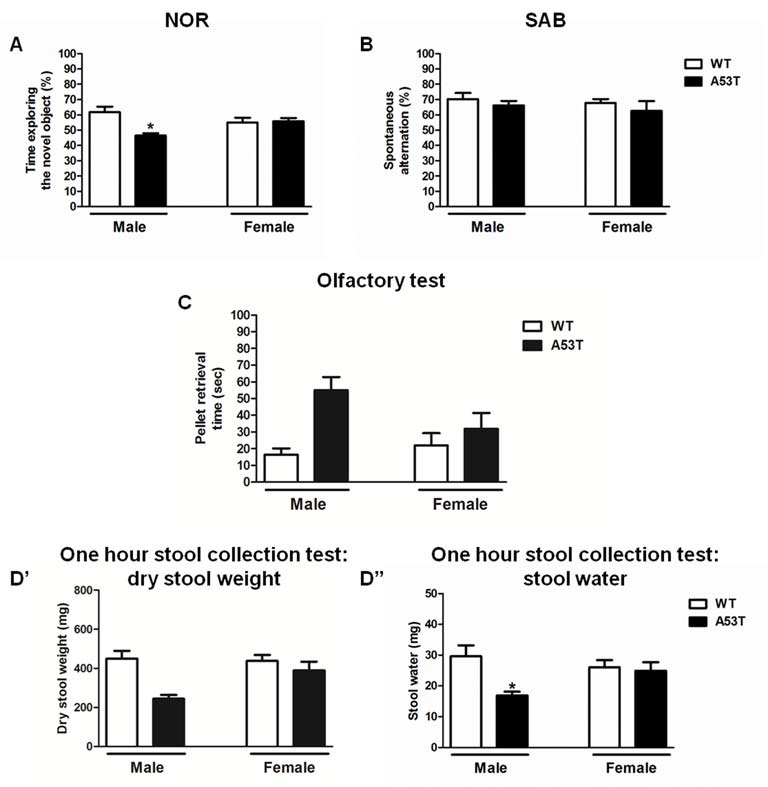
Memory and peripheral tasks in male and female A53T transgenic and WT mice. Representative histograms for the novel object recognition (NOR; **A**), spontaneous alternation behavior in a Y-maze (SAB; **B**), olfactory **(C)** and 1-h stool collection **(D’,D”)** tests performed in male and female A53T transgenic and WT mice. Data in **(A)** show the mean ± SEM of the percentage of time spent exploring the novel object. Data in **(B)** show the mean ± SEM of the percentage of spontaneous alternation. Data in **(C)** show the mean ± SEM of the time required to retrieve a pellet (sec). Data in **(D’)** show the mean ± SEM of the dry stool weight (mg), whereas data in **(D”)** show the mean ± SEM of the stool water content (mg). The number of mice per group in **(A)** is as follows: male A53T transgenic mice *n* = 5; male WT mice *n* = 5; female A53T transgenic mice *n* = 11; female WT mice *n* = 14. The number of mice per group in **(B)** is as follows: male A53T transgenic mice *n* = 6; male WT mice *n* = 7; female A53T transgenic mice *n* = 11; female WT mice *n* = 14. The number of mice per group in **(C)** is as follows: male A53T transgenic mice *n* = 8; male WT mice *n* = 7; female A53T transgenic mice *n* = 11; female WT mice *n* = 12. The number of mice per group in **(D’,D”)** is as follows: male A53T transgenic mice *n* = 4; male WT mice *n* = 7; female A53T transgenic mice *n* = 12; female WT mice *n* = 14. *p < 0.05 compared with WT mice by Newman–Keuls *post hoc* test.

Two-way ANOVA revealed a significant effect of genotype (*F*_(1,31)_ = 4.33, *p* < 0.05), and a significant gender × genotype interaction (*F*_(1,31)_ = 5.57, *p* < 0.05), but no significant effect of gender (*F*_(1,31)_ = 0.12, *p* > 0.05; Figure 6A). The Newman–Keuls *post hoc* test indicated that male A53T transgenic mice had an impaired NOR performance compared with male WT mice (*p* < 0.05), whereas female A53T and WT mice displayed a comparable NOR performance (*p* > 0.05). In all the NOR experiments performed, no significant differences in the total amount of time spent exploring the objects during S1 and S2 were observed among the various experimental groups (data not shown).

#### SAB Task

Two-way ANOVA revealed no significant effect of genotype (*F*_(1,34)_ = 0.93, *p* < 0.05) and gender (*F*_(1,34)_ = 0.42, *p* < 0.05), as well as no significant gender × genotype interaction (*F*_(1,34)_ = 0.01, *p* < 0.05; Figure 6B). Moreover, no significant differences in the numbers of entries into the arms of the Y-maze were observed among the various experimental groups (data not shown).

#### Olfactory Test

Two-way ANOVA revealed a significant effect of genotype (*F*_(1,34)_ = 8.62, *p* < 0.01) but neither a significant effect of gender (*F*_(1,34)_ = 1.13, *p* > 0.05) nor a significant gender × genotype interaction (*F*_(1,34)_ = 2.99, *p* > 0.05; Figure 6C). The Newman–Keuls *post hoc* test indicated that A53T transgenic mice took a significantly longer time to retrieve the pellet, compared with WT mice (*p* < 0.05), and this effect appeared to be more pronounced in male than in female mice.

#### One-Hour Stool Collection

For the stool dry weight, two-way ANOVA revealed a significant effect of genotype (*F*_(1,33)_ = 7.80, *p* < 0.01), but neither a significant effect of gender (*F*_(1,33)_ = 2.15, *p* > 0.05) nor a significant gender × genotype interaction (*F*_(1,33)_ = 2.95, *p* > 0.05; Figure 6D′). The Newman–Keuls *post hoc* test indicated that A53T transgenic mice had a lower stool dry weight, compared with WT mice (*p* < 0.05), and this effect appeared to be more pronounced in male than in female mice.

For the stool water content, two-way ANOVA revealed a significant effect of genotype (*F*_(1,33)_ = 5.71, *p* < 0.05), and a significant gender × genotype interaction (*F*_(1,33)_ = 3.95, *p* < 0.05), but no significant effect of gender (*F*_(1,33)_ = 0.58, *p* > 0.05; Figure 6D″). The Newman–Keuls *post hoc* test indicated that male A53T transgenic mice had a lower stool dry weight, compared with male WT mice (*p* < 0.05), whereas female A53T and WT mice displayed a comparable stool weight (*p* > 0.05).

## Discussion

A53T transgenic male mice spontaneously develop several phenotypical and neurochemical abnormalities that resemble those of PD pathology, including motor deficits, nigrostriatal dopaminergic degeneration with signs of α-synuclein aggregation, and gliosis (Giasson et al., 2002; Martin et al., 2006; Sirabella et al., 2018). Although earlier studies have described motor deficits, dopamine neuron degeneration, neuroinflammation and mitochondrial dysfunctions in A53T transgenic male mice (Martin et al., 2006; Unger et al., 2006), limited information is currently available on the alterations that involve these parameters in this strain, especially in female animals. Moreover, a previous investigation by our group (Sirabella et al., 2018) has demonstrated that male A53T transgenic mice have reduced expression and activity of NCX3 in the midbrain, but display an increased expression and activity of NCX1 in the striatum together with a co-expression of NCX3 in TH-positive dopamine neurons in the SNc, and a co-expression of NCX1 in IBA-1-positive microglial cells in the striatum. Also, TH-positive nigral neurons and striatal fibers were decreased in male A53T transgenic mice, and these effects were accompanied by an increase of GFAP-positive astroglial cells in the SNc and striatum, as well as of IBA-1-positive cells in the striatum. Taken together, these data led us to conclude that abnormalities in the NCX1 and NCX3, which regulate cytosolic and mitochondrial Ca^2+^ homeostasis in the midbrain and striatum, might play a role in the dopaminergic nigrostriatal degeneration and in the neuroinflammation that occurs in 12-month-old male A53T transgenic mice (Sirabella et al., 2018).

In continuity with those earlier findings, and with the scope of evaluating the presence of gender differences as described in human PD, the present study provides new insights into the relevance of A53T transgenic mice as a model of PD, by demonstrating that female mice exhibit a limited PD-like phenotype and dissimilar regional distribution of NCX1 and NCX3 from that previously described in male mice (Sirabella et al., 2018). Indeed, in contrast to earlier findings in male A53T transgenic mice (Sirabella et al., 2018), the present study found that 12-month-old female A53T transgenic mice displayed a lack of co-localization of NCX3 with TH-positive neurons, similarly to what observed in female WT mice. This finding may be consistent with the other results of the present study showing that 12-month-old female A53T transgenic mice have a decreased number of TH-positive neurons in the SNc. Earlier investigations in models of neurodegeneration have suggested that activation of NCX3 may prevent neuronal damage, as shown by the evidence that the neurotoxic effects of amyloid Aβ_1–42_ are exacerbated in NCX3-silenced neurons (Pannaccione et al., 2012). Hence, the results of this study would suggest that the regional distribution of NCX3 in regions of the brain of A53T transgenic mice may vary with gender and possibly be correlated to the observed neurodegeneration.

At variance to the decrease of TH observed for nigral neurons, female A53T transgenic mice displayed no degeneration of dopaminergic fibers in the striatum, which differs from previous observations in male mice of the same strain, in which TH immunoreactivity was decreased in both the SNc and striatum (Sirabella et al., 2018). It must be considered, however, that dopaminergic degeneration is usually more pronounced in the SNc than in the striatum, as demonstrated by previous studies that employed neurotoxic substances (Frau et al., 2016; Noël et al., 2018), which may suggest that female A53T transgenic mice have a dopamine neuron degeneration of lower intensity compared with males. We, therefore, speculate that gender differences exist in A53T transgenic mice concerning the nigrostriatal dopaminergic system degeneration and that these differences may eventually result in a loss of dopaminergic striatal fibers in male but not female animals. Regarding NCX3 we might also speculate that the exchanger contributes to the activation of glial cells, with consequent release of pro-inflammatory factors, which in turn might sustain the dopaminergic neurodegeneration in male A53T transgenic mice (Boscia et al., 2013). The finding of the present study showing no presence of NCX3 in the SNc of A53T transgenic female mice may lead to hypothesize that such a lack of NCX3 could explain the gender-dependence of dopaminergic degeneration observed in A53T transgenic mice, and the sparing of striatal dopaminergic terminals in female, but not male, animals.

Another finding of this study is that, similarly to what observed in 12-month-old male A53T transgenic mice, NCX1 co-localized with IBA-1-positive microglial cells in the striatum but not SNc of 12-month-old female A53T transgenic mice. In contrast, neither microgliosis nor astrogliosis was observed in the nigrostriatal system of A53T transgenic female mice, which is at odds with earlier findings showing marked microgliosis and astrogliosis in the striatum of male mice of the same strain (Sirabella et al., 2018). The lack of co-localization between IBA-1 and NCX1 observed here in the SNc may suggest that gliosis and other mechanisms of toxicity that may be mediated by NCX1 are minimally involved in the degeneration of nigral dopaminergic neurons that occurs in female A53T transgenic mice. This hypothesis may support the possibility that the mechanisms that promote and sustain nigrostriatal dopaminergic degeneration differ between male and female A53T transgenic mice.

A previous study has demonstrated that an upregulation of NCX1 expression and activity occurs in the cortex of ischaemic rats subjected to the occlusion of the medial cerebral artery, leading to the hypothesis that activation of NCX1 has a protective role in the brain (Boscia et al., 2009). On this basis, the results of this study support a possible neuroprotective role of NCX1 co-localized in IBA1 neurons in the striatum, a region that is not affected by dopamine terminal degeneration and does not show any glial activation. Moreover, these results strengthen the relevance of A53T mice as a model with which to study how different factors that have been associated with an increased likelihood of developing PD (i.e., mitochondrial dysfunction, neuroinflammation) may be involved in the demise of dopaminergic neurons and the manifestation of PD-like phenotypes.

In our previous study (Sirabella et al., 2018), we found that male A53T transgenic mice displayed motor deficits in the beam walking, pole and open field tests, which are paradigms suited to revealing in rodents the presence of phenotypical alterations that resemble those found in PD. On this basis, we evaluated the performance of 12-month-old female A53T transgenic mice in the beam walking and pole tests, to ascertain whether dopaminergic nigrostriatal degeneration observed in these animals was paralleled by altered motor function. In the beam walking test, female A53T transgenic mice required a longer time to cross the beam, compared with WT mice. However, the number of steps and the errors per step were comparable between female A53T transgenic and WT female mice. Moreover, female A53T transgenic and WT mice displayed a similar performance in the pole test. Taken together, these findings indicate that female A53T transgenic mice develop only mild motor impairment, which appears consistent with the results of immunohistochemistry showing that the same mice displayed only partial degeneration of the nigrostriatal tract.

The presence of behavioral and phenotypical abnormalities in 12-month-old female A53T transgenic mice was also evaluated using non-motor tasks, in parallel with the same evaluation in 12-month-old male mice, to further elucidate whether gender had any influences on the phenotype of A53T transgenic mice. Male A53T transgenic mice displayed an impaired performance in the NOR task, which is indicative of a deficit in the non-spatial component of short-term memory, but showed no alterations in SAB, which suggests that these animals had a normal general cognitive function and spatial memory. These discrepant results could be explained considering previous studies which showed that SAB may be relatively resistant to noxious brain insults that impair NOR performance, suggesting a different sensitivity of these two tasks to memory impairment (Simola et al., 2008; Moriguchi et al., 2012; Costa et al., 2014). Moreover, compared to WT mice, male A53T transgenic mice displayed a trend towards an increase in the amount of time required to retrieve an odorous pellet, which would indicate an abnormal olfactory function. Also, they displayed a reduced water content in the stool, which is an index of constipation. Interestingly, female A53T transgenic mice showed no overt abnormalities in the above tests. Since memory deficits, olfactory dysfunction and constipation are non-motor symptoms that are present together with motor impairment in PD patients (Postuma et al., 2012), our findings would suggest that female A53T transgenic mice exhibit a less pronounced PD-like phenotype, compared with male mice of the same strain.

Importantly, the behavioral and phenotypical markers examined in this study are all dependent on dopaminergic functions and are influenced by neuroinflammation (Barnum and Tansey, 2012; Kim et al., 2015). Therefore, we may conclude that the low dopamine neuron degeneration and the absence of neuroinflammation may explain why A53T female mice displayed modest motor and non-motor deficits.

In conclusion, the findings of low vulnerability to striatal dopaminergic degeneration and nigrostriatal neuroinflammation in female A53T transgenic mice further increase the interest in this mouse strain as a model suitable for use in preclinical studies of gender differences in PD and lead us to hypothesize that NCX1 and NCX3 play a role in determining the different pattern of dopaminergic degeneration that can be observed in the nigrostriatal systems of male and female A53T transgenic mice.

## Data Availability Statement

The datasets generated for this study are available on request to the corresponding author.

## Ethics Statement

The animal study was reviewed and approved by Organismo per il benessere degli animali (OPBA) University of Cagliari.

## Author Contributions

GC, MC, MJS, and SD performed the behavioral and biochemical experiments. GC, MJS and SD analyzed the data. MJS and AS bred the mice colony. GC, NS, AS and MM wrote the manuscript. GC, NS, AP, MS, AS and MM reviewed and edited the manuscript. GC, MJS, NS, SD, MC, MS, AP, AF, LA, AS and MM read and approved the final manuscript.

## Conflict of Interest

The authors declare that the research was conducted in the absence of any commercial or financial relationships that could be construed as a potential conflict of interest.

## Abbreviations

ABC: avidin-biotin-peroxidase complex
ANOVA: analysis of variance
DAPI: 4′,6-diamidine-2′-phenylindole dihydrochloride
GFAP: glial fibrillary acidic protein
IBA-1: ionized calcium binding adaptor molecule 1
NCX: Na^+^-Ca^2+^ exchanger
NOR: novel object recognition
PD: Parkinson’s disease
Rr: Pearson correlation coefficient
SAB: spontaneous alternation behavior in a Y-maze
SNc: substantia nigra pars compacta
TH: tyrosine hydroxylase
WT: wild type.
